# Mutation analysis of *Rad18 *in human cancer cell lines and non small cell lung cancer tissues

**DOI:** 10.1186/1756-9966-28-106

**Published:** 2009-07-25

**Authors:** Tadahiko Nakamura, Shinji Ishikawa, Yoshikatsu Koga, Youhei Nagai, Yu Imamura, Kouei Ikeda, Takeshi Mori, Hiroaki Nomori, Hideo Baba

**Affiliations:** 1 Department of Gatroenterological Surgery, Graduate School of Medical Sciences, Kumamoto University, 1-1-1 Honjo, Kumamoto 860-8556, Japan; 2 Department of Thoracic Surgery, Graduate School of Medical Sciences, Kumamoto University, 1-1-1 Honjo, Kumamoto 860-8556, Japan

## Abstract

**Background:**

Genetic instability is known as a cause of oncogenesis. Though Rad18 is reported to function in a post replication mismatch repair system, the relation between the status of Rad18 and human tumorigenesis has not been described so far.

**Methods:**

Mutation analysis of 34 human cancer cell lines and 32 non small cell lung cancer (NSCLC) tissues were performed by RT-PCR SSCP. Expression level of *Rad18 *was measured by real time RT-PCR. Stable transfectant was constructed for in vitro study.

**Results:**

No mutation was found in both cancer cell lines and NSCLC tissues. A single nucleotide polymorphism (SNP) at codon 302 was detected in 51.5% of the cell lines and 62.5% of NSCLC tissues. Interestingly, *Rad18 *was homozygously deleted in a pulmonary adenocarcinoma cell line PC3. Furthermore, there was no difference in the expression level of wild type *Rad18 *and *Rad18 *with SNP. The growth, cell morphology, sensitivity to anti-cancer drugs and in vitro DNA repair activity between wild type *Rad18 *and *Rad18 *with SNP revealed to have no difference in vitro.

**Conclusion:**

Though the frequency of SNP was tended to be higher in NSCLC patients than healthy volunteers (57.7%), as the difference was not significant, we have concluded that there is no relation between *Rad18 *SNP and lung cancer development.

## Background

Endogenous and environmental factors such as ultraviolet, ionizing radiation, and numerous genotoxic chemicals can cause DNA damage. These DNA lesions can be repaired by various repair mechanisms [1]. For example, the most frequent DNA lesions are efficiently removed by pathways called base excision repair and nucleotide excision repair [2]. However, some unrepaired DNA lesions can remain at replication because of limited capacity of DNA repair systems. These lesions induce gaps in the newly synthesized strand. The gaps are filled by postreplication repair (PRR) system and this repair system is conserved from yeast to mammalian cells [3,4]. In the yeast Saccharomyces cerevisiae, genes belonging to the Rad6 epistasis group play an important role in the PRR pathway [5]. In this pathway, Rad6 and Rad18 are the most important genes. Rad6 is an ubiquitin-conjugating enzyme (E2) and Rad18 is a single-stranded DNA binding protein and has ubiquitin-ligase (E3) activity. Rad18 forms a specific complex with Rad6 [6,7]. Human homolog of yeast *Rad18 *gene is mapped on chromosome 3p24-25 and it has been shown that human Rad18 protein interacts with the human homologs of the Rad6 protein (HHR6A and HHR6B) and is involved in PRR [8,9]. Rad18 or Rad6 mutations cause higher sensitivity to various mutagens [10]. Inactivation of Rad18 in mouse embryonic stem cells leads to increasing sensitivity to various DNA-damaging agents and to increasing sister-chromatic exchange. Rad18 contributes to maintenance of genomic stability through PRR [10]. However, the status of *Rad18 *in human cancers is still unknown.

In the present study, we analyzed the expression and the mutation of *Rad18 *in human cancer cell lines and NSCLC tissues and also assessed whether there is some functional difference due to the SNP of *Rad18*.

## Methods

### Cell lines and cell culture

Twenty-nine digestive carcinoma cell lines and five lung carcinoma cell lines were used in this study. They comprised: 7 esophageal carcinoma cell lines (KYSE30, KYSE140, TE1, TE9, TE10, TE12, TE13), 6 gastric carcinoma cell lines (AGS, MKN1, MKN28, MKN45, NUGC3, NUGC4), 9 colon carcinoma cell lines (Caco2, Colo201, Colo205, DLD-1, HCT116, HT29, SW480, SW620, WiDr), 7 pancreatic carcinoma cell lines (AsPC-1, Capan1, Capan2, Panc1, SUIT-2, MiaPaCa2, Hs700T) and 5 lung carcinoma cell lines (A549, EBC1, LU99, PC3, LCOK). Cell lines were cultured in recommended medium supplemented with 10% fetal bovine serum (Invitrogen) at 37°C in a humidified atmosphere of 5% CO_2 _to 95% air.

### Tissue samples

Non-small cell lung cancer samples were all surgically resected in Kumamoto University Hospital (Kumamoto, Japan) between 2005 and 2006. Informed consent was performed to all patients. Only the samples with agreement were used for further analysis. This study was approved by the ethical committees of Kumamoto University Hospital. The following features were looked at: sex, age, and pathological status (size, histological type, T stage, lymph node metastasis, pStage). UICC Tumor-Node-Metastasis Classification of Malignant Tumors [11] was used to classify pathological status. For the controls, peripheral white blood cells of 26 healthy volunteers were collected.

### RNA isolation

The frozen tissue samples were ground into powder in liquid nitrogen. Total RNA of tissue samples and cell lines were isolated by using Trizol reagent according to the instruction manual (Invitrogen). Total RNA of leukocytes obtained from 2 ml of peripheral blood was isolated by using PURESCRIPT RNA Isolation Kit (Gentra systems).

### RT-PCR

Five microgram of the total RNA was reverse transcribed using oligo-dT primer and SuperScript III (Invitrogen) according to the instruction manual. To confirm the expression of *Rad18*, primer sets, 5'-TTC, ACA, AAA, GGA, AGC, CGC, TG (forward) and 5'-TTA, CTG, AGG, TCA, TAT, TAT, CTT, C (reverse) were used to amplify 310 bp region of human *Rad18 *gene. PCR was carried out in a condition of, 3 min at 94°C for initial denaturing, followed by 35 cycles of amplification (94°C for 30 sec, 55°C for 30 sec, and 72°C for 30 sec) using GoTaq (Promega). The amplified products were visualized on 1.2% agarose gel with ethidium bromide. GAPDH in the same samples was also amplified using 25 cycles PCR reaction as the internal control. The primer sets for GAPDH is 5'-TGA, CCA, CAG, TCC, ATG, CCA, TC (forward) and 5'-CCA, CCC, TGT, TGC, TGT, AGC, C (reverse).

### Fragment Southern

Genomic DNA from human breast cancer cell line MCF7 and lung carcinoma cell line PC3 were isolated using TRIZOL according to the instruction manual. MCF7 was used as positive control which was confirmed that this cell line carry wild type *Rad18 *by RT-PCR direct sequencing (data not shown). Ten microgram of genomic DNA were digested by EcoRI or HindIII, electrophoresed on a 0.8% agarose gel and transferred to a Hybond-NX membrane (Amersham). Full length cDNA clone of *Rad18 *was labeled using Psoralen-Biotin nonisotopic labeling kit (BrightStar) and hybridized in PEG-SDS including 100 μg/ml Salmon sperm DNA at 65°C. Detection was done using BioDetect nonisotopic detection kit (BrightStar) according to the instruction manual. Membrane was exposed to X-ray film and developed.

### RT-PCR SSCP and direct sequencing

The primer sets for RT-PCR SSCP are shown in Table 1. Each primer sets were designed to partially overlap the next fragment with the length not more than 200 bp. Ten primer sets cover the whole open reading frame of *Rad18 *gene and partially, 5' and 3' non coding lesion. PCR condition is, 3 min at 94°C for initial denaturing, followed by 35 cycles of amplification (94°C for 30 sec, 55°C for 30 sec, and 72°C for 30 sec). Each sample was denatured 5 min at 95°C and rapidly chilled on ice and loaded into 10% acrylamide gel including 5.4% glycerol for 6 hours at 120V using MiniProtean3 (BioRad) at 4°C. After electrophoreses, gels were stained using Silver Stain Plus Kit according to the instruction manual (BioRad). All samples were screened for the presence of an aberrant band compared with reference sample. Samples with abnormal SSCP bands were directly sequenced by ABI 310. Cycle sequencing was performed using Big-Dye Terminator v3.1 (Applied Biosystems). Sequencing analysis was done using Sequencing Analysis Software (Applied Biosystems).

**Table 1:** Primer sets for Rad 18 RT-PCR SSCP

No.	Forward	Reverse
1.	CAG,CAT,CCT,CGG,GAG,CG	AGG,ACA,GAA,ATT,TTC,TTA,TAC,AG
2.	CCT,CAG,TGT,TCA,CAT,AAC,TAC	GGA,GAT,TTG,GCT,GGT,GAC,TC
3.	ACG,GAA,TCA,TCT,GCT,GCA,GT	TTT,TAT,TTT,CTT,TTA,TCA,ACA,ACT,C
4.	AGA,AAT,GAG,TGG,TTC,TAC,ATC,A	GAC,AAT,CCA,CTT,TAGT,AAC,TTG
5.	TCC,TGA,GCC,ACC,CTC,GAC	ATC,AGA,GAG,CAA,ATT,ATA,TAC,AG
6.	TTC,ACA,AAA,GGA,AGC,CGC,TG	CTT,GAA,CTA,TTT,CAG,CAG,CTG
7.	TAC,AAT,GCC,CAA,TGC,GAT,GC	AAA,TTC,ACT,CTT,ATG,TTT,TTT,ACG
8.	AGG,AAA,TAG,ATG,AAA,TCC,ACA,G	TTA,CTG,AGG,TCA,TAT,TAT,CTT,C
9.	AGC,TAT,CTT,CTG,TATG,CAT,GG	CTC,TTA,TGA,TGT,CTG,AAC,TGG
10.	CAG,AAT,CAG,ATT,CAT,GCA,ATA,G	AAG,TCA,GCA,AAA,GCC,CAC,ATT

### Real time-PCR

Complimentary DNA, primers (10 pmol/μl) and Hybprobe probes (10 pmol/μl) were mixed in the LightCycler FastStart DNA Master HybProbe Kit according to the instruction manual (Roche Diagnostics). The primers and probes are as follow: forward primer 5'-AGC, CTG, GGA, AGC, ATC, ACA, TA, reverse primer 5'-CTG, TGG, CAA, CCA, AAA, GTA,CG, Fluorescein probe 5'-CGC, TGA, AAG, TGC, TGA, GAT, TGA, ACC, AAG, AA, LCRed640 probe 5'-CAA, GCG, TAA, TAG, GAA, TTA, ATG, TGG, GCT, TTT, GC. PCR was carried out in the LightCycler System (Roche Diagnostics). Cycling conditions were 1 cycle of 95°C for 10 minutes, 40 cycles of amplification (95°C for 10 sec, 62°C for 10 sec, 72°C for 6 sec). The concentration of GAPDH in the same samples was also quantified using the LightCycler-Primer Set (Nihon Gene). The concentration of *Rad18 *was calculated as a ratio to the amount of GAPDH detected.

### Cloning of *Rad18*

Full length of *Rad18 *were amplified using primer sets, 5'-ATT, TCG, AGT, GGT, GTT, GGA, GC (forward) and 5'-TGG, TAC, CTG, TGT, GAA, ATG, TC (reverse). MCF7 cDNA was used as a template for wild type *Rad18 *and EBC1 cDNA for SNP *Rad18*. Each product was ligated into plasmid vector pcDNA3.1/V5-His-TOPO (Invitrogen). Clones were sequenced using ABI310 and confirmed for no PCR error.

### Construction of stable transfectant

The PC3 cell line were transfected with either wild type *Rad18 *or *Rad18 *SNP, using lipofectamine2000 (Invitrogen). Stable transfectants were selected for 4 weeks in Dulbecco's Modified Eagle Medium (GIBCO) containing G418 (400 μg/ml). We designated PC3 cell line with wild type *Rad18 *as PC3-WT *Rad18 *and PC3 cell line with *Rad18 *SNP as PC3-SNP *Rad18*. PC3 cell line transfected with pcDNA LacZ was also constructed as a control.

### Cell growth and cell survival assay

Prior to the day before experiment, 5 × 10^4 ^of PC3-WT *Rad18 *and PC3-SNP *Rad18 *cells were plated on a twelve-well plate and incubated at 37°C. For growth assay, cells were counted using hemocytometer at day 1, 3, 5, 7. For cell survival assay, 5 × 10^4 ^cells per well were plated on a twelve-well plate and indicated dose of cisplatin or CPT-11 were added to the medium from day 1. Culture medium containing cisplatin or CPT-11 was changed daily. At day1, 3, 5, 7, the cells were washed twice with PBS, trypsinized and stained by Trypan Blue solution. Living cells were counted using hemocytometer. All measurements were performed in triplicate.

### Western Blotting

Whole cell lysate from PC3-LacZ, PC3-WT Rad18, and PC3-SNP Rad18 were extracted using RIPA buffer including protease inhibitor and phosphatase inhibitor. Twenty-five micrograms of whole cell lysate were electrophoresed in 10% SDS-PAGE gels and transferred on to PVDF membrane. The membranes were blocked with 5% NFDM in PBS/Tween20 (0.1%) at room temperature for 1 hour and then were incubated with Rad18 first antibody (Santa Cruz) for 1 hr at room temperature. The membrane was then washed for 10 min 2× with PBS/Tween20 and then were incubated with anti goat IgG second antibody (Santa Cruz) for 45 min at room temperature. The membranes were washed for 10 min 2× with PBS/Tween20 and for 10 min 1× with PBS, incubated with ECL-Plus and then were exposed to X-ray film and developed.

### In vitro DNA repair assay

The activity of DNA repair was measured using RPA DNA repair kit (Active Motif) according to the instruction manual. PC3 cells were plated on a 6 well plate the day before transfection. Three micro grams of LacZ, WT Rad18, Rad18 SNP and the mixture of 1.5 μg each of WT and SNP Rad18 plasmid were transfected to the cells as described above. Forty hours after transfection, the cells were irradiated by UV for 30 sec to damage DNA, and the nuclear extract were purified 48 hr after transfection according to the instruction manual. Various dose of the nuclear extract (1 to 5 μg) were added to the 96 well plate provided by the kit and reacted. The absorbance was read using kinetic microplate reader V-max (Molecular Devices). All measurements were performed in triplicate. Values of *P *< 0.05 were considered to be statistically significant.

## Results

### Expression of *Rad18 *in human cancer cell lines

**Figure 1:**
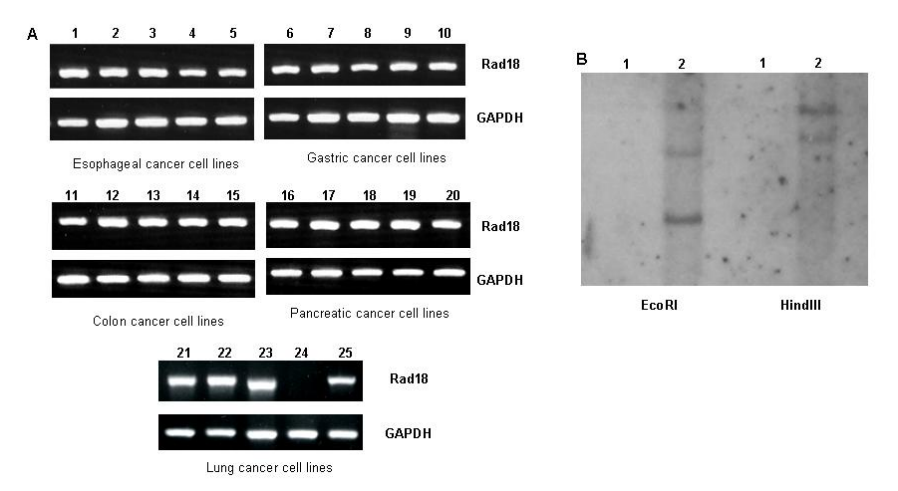
**The expression of *Rad18 *in human cancer cell lines**. A: RT-PCR analysis of *Rad18 *in human cancer cell lines. A part of cell lines examined are present. The expression of *Rad18 *mRNA is observed in all cancer cell lines but PC3 (lane 24). Lane 1: KYSE30, 2: KYSE140, 3: TE1, 4: TE9, 5: TE10, 6: AGS, 7: MKN1, 8: MKN28, 9: NUGC3, 10: NUGC4, 11: Caco2, 12: Colo201, 13: Colo205, 14: DLD-1, 15: HCT116, 16: AsPC-1, 17: Capan1, 18: Capan2, 19: Panc1, 20: SUIT-2, 21: A549, 22: EBC1, 23: LU99, 24: PC3, 25: LCOK. B: Fragment Southern of PC3 (lane 1) and MCF7 (lane 2). Rad18 is homozygously deleted in lung cancer cell line PC3.

The expression of *Rad18 *gene in human cancer cell lines was analyzed by RT-PCR. Except for PC3 cell line, *Rad18 *gene was expressed in all digestive and lung cancer cell lines (Figure 1A). In PC3, no amplification was observed also in PCR using PC3 genomic DNA as a template (data not shown). Fragment southern blotting revealed that the genomic lesion of *Rad18 *was homozygously deleted in PC3 lung cancer cell line (Figure 1B).

### Mutational analysis of *Rad18 *in cancer cell lines

**Figure 2:**
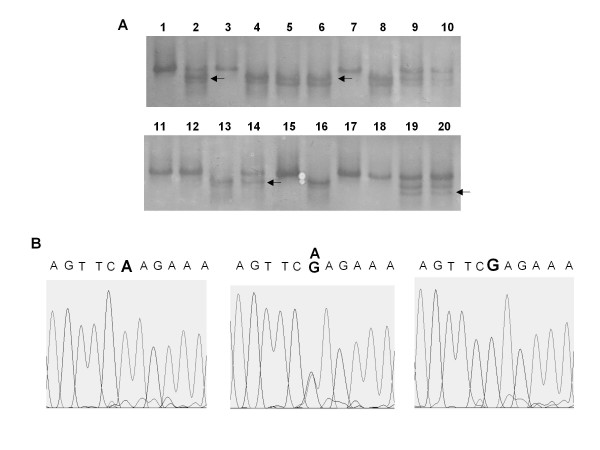
**SSCP analysis of human cancer cell line**. A: A part of SSCP of primer set 7 is present. The shifted abnormal band is pointed. B: The result of direct sequence of the shifted band. At codon 302, three different patterns were detected.

RT-PCR SSCP of *Rad18 *in 33 cancer cell lines was analyzed by PCR-SSCP. Within the ten PCR fragment, only PCR fragment No.7 showed abnormal band pattern in 17 cell lines (Figure 2A). Direct sequence of the abnormal band revealed that all 17 cell lines carried a single nucleotide polymorphism (SNP) at the second letter of codon 302 (51.5%) and there were no other mutation (Figure 2B). This SNP was already reported in the SNP database. Though there was no coding region mutation, as lung cancer cell line PC3 had a homozygous deletion in *Rad18 *genomic lesion and as *Rad18 *is mapped at chromosome 3p25 which is reported to have frequent LOH in lung cancer [12], we decided to analyze lung cancer tissue for *Rad18 *mutation.

### Mutation analysis of *Rad18 *in NSCLC tissues

**Figure 3:**
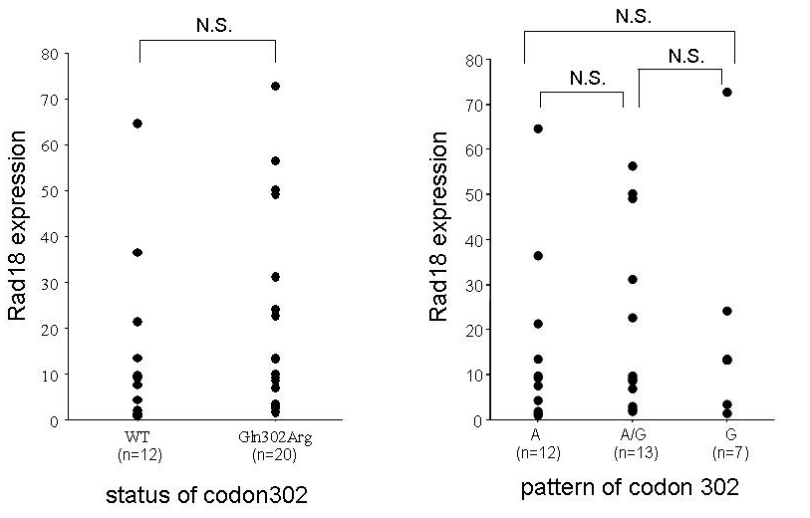
***Rad18 *expression level in lung cancer tissues**. Left: Expression level according to wild type and SNP. Right: Expression level according to the pattern of codon 302.

The clinicopathological characteristics of examined NSCLC patients are shown in Table 2. First we checked the expression of *Rad18 *by RT-PCR The expression of *Rad18 *was observed in all 32 NSCLC tissues. RT-PCR SSCP revealed that there was no mutation in *Rad18 *coding region but the same SNP of codon 302. This SNP was observed in 20 samples of 32 NSCLC tissues (62.5%) and in 15 peripheral blood samples of 26 healthy volunteers (57.7%). Though, the frequency of *Rad18 *SNP is tended to be higher in NSCLC tissue than the healthy volunteers, the difference was not significant. There was no difference in other characters such as sex, histological type, T-stage, lymph node metastasis or p-stage between WT and SNP (Table 2). In addition, there were no difference between the three patterns of codon 302 and lung cancer development (Table 3). Furthermore, *Rad18 *expression level was also examined using light cycler (Fig 3). No difference was observed between WT and SNP or between the three patterns of codon 302.

**Table 2:** Clinicopathological characteristics of NSCLC

	WT (n = 12)	SNP (n = 20)	
			N.S.
Age (years, mean ± SD)	70.6 ± 8.2	70.0 ± 8.8	
			N.S.
Sex			
Male	9	12	
Female	3	8	
			N.S.
Histological type			
Squamous cell carcinoma	7	3	
Adenocarcinoma	3	14	
Others	2	3	
			N.S.
T stage			
T1	4	13	
T2	7	7	
T3	1	0	
			N.S.
Lymph node metastasis			
Positive	2	4	
Negative	10	16	
			N.S.
pStage			
IA	4	11	
IB	3	5	
IIA	0	2	
IIB	5	2	

**Table 3:** Frequency of Rad18 Gln302Arg polymorphism

	Lung cancer tissue	Healthy volunteers	
No. of samples	32	26	
			
No. of polymorphism	20 (62.5%)	15 (55.7%)	N.S.
			
Pattern of codon 302			N.S.
A (Gln)	12 (37.5%)	11 (42.3%)	
A/G (Gln/Arg)	13 (40.7%)	7 (26.9%)	
G (Arg)	7 (21.9%)	8 (30.8%)	
			
Frequency			
A (Gln)	37 (57.8%)	29 (55.8%)	N.S.
G (Arg)	27 (42.2%)	23 (44.2%)	

### In vitro study of Rad18 polymorphism

**Figure 4:**
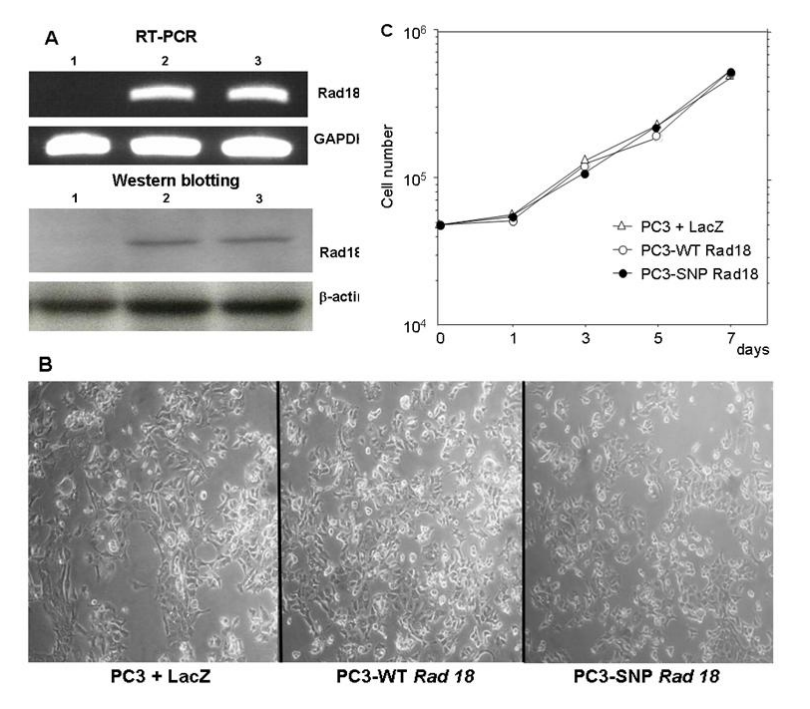
**In vitro study of *Rad18 *WT and *Rad18 *SNP**. A: Expression of introduced Rad18 assessed by RT-PCR (top) and Western blotting (bottom). Lane 1: PC3 + LacZ, 2: PC3-WT *Rad18*, 3: PC3-SNP *Rad18*. B: Cell morphology of the three cell lines. C: Growth assay of the three cell lines. D: Sensitivity to CDDP (left) and CPT-11 (right) in the three cell lines. E: Percent survival at day 7 for different dose of CDDP (left) and CPT-11 (right).

**Figure 5:**
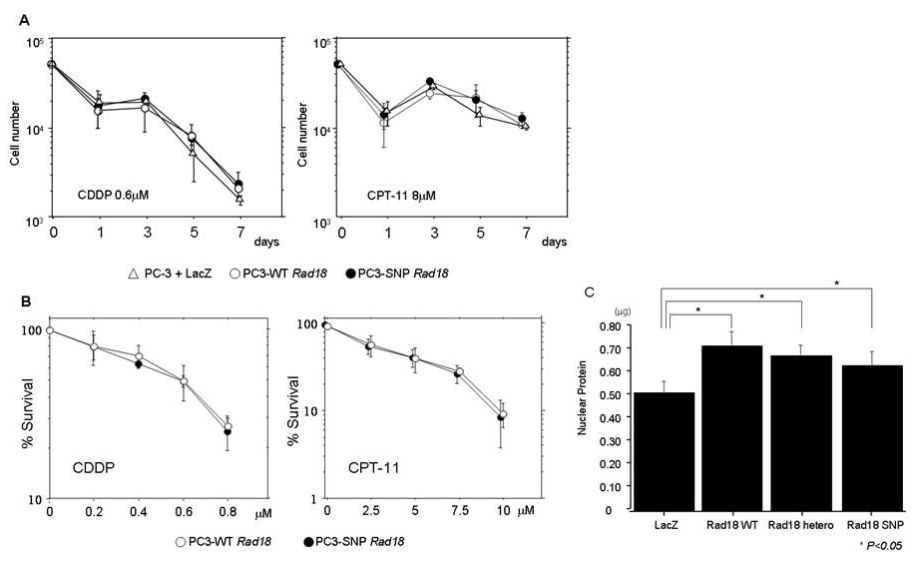
**Drug sensitivity and repair function of Rad18 and the SNP**. A: Sensitivity to CDDP (left) and CPT-11 (right) in the three cell lines. B: Percent survival at day 7 for different dose of CDDP (left) and CPT-11 (right). C: DNA repair assay of LacZ, WT(A/A), hetero(A/G), SNP(G/G). The vertical axis is the amount of RPA protein which shows the activity of DNA repair function.

Though there was no *Rad18 *mutation in human cancer cell line and NSCLC tissue examined except PC3, as Rad18 functions as post-replication repair system, we have examined whether there is any difference between wild type Rad18 and Rad18 SNP in vitro. Using Rad18 null cell line PC3, wild type *Rad18 *or *Rad18 *SNP was transfected. The expression of introduced *Rad18 *gene was confirmed by RT-PCR and Western blotting (Fig 4A). The cell morphology of these stable transfectant had no difference (Fig 4B). Additionally, there was no difference in growth, sensitivity or survival rate against anti-cancer drugs (CDDP or CPT-11) (Fig 4C, 5A, B). Furthermore, the in vitro DNA repair showed that, when PC3 was transfected with Rad18, the DNA repair was induced compared to the control (LacZ transfected PC3). However, there was no difference between the status of the codon 302 (A/A, A/G, G/G) (Fig 5C).

## Discussion

There is no doubt that genetic instability is one of the main causes of cancer development. Genetic instability can be divided in two. One is chromosomal instability and the other is microsatellite instability (MSI). It is reported that chromosomal instability is frequently found in lung cancer but microsatelite instability is rare [13]. Though 60% of non small cell lung cancer has loss of heterozygosity (LOH) in 3p and it is suggested that several tumor suppressor genes might be mapped in this region, a clear relation between lung cancer development and a single gene mutation has not been reported to date [14,15]. Concerning microsatellite instability, using microsatellite markers located at 3p or targeting human mismatch repair gene, hMLH1, has been analyzed [16,17]. They concluded that MSI is not frequently found in lung cancer tissue or pleural effusion of lung cancer patients.

We focused on Rad18 which functions as a PRR system and mapped on 3p25. Within the cell lines and lung cancer tissues that we examined, no *Rad18 *mutation was detected but a homozygous deletion in PC3 (lung cancer cell line). This result suggests that there might be no relation between Rad18 mutation and lung cancer development. During the SSCP analysis, we found a SNP (Gln 302 Arg) which was relatively frequent in lung cancer tissues. Recently, a report that the same SNP of *Rad18 *is associated to the risk of lung cancer was published [18]. Different to our study, this report was focused only on the SNP and the mutation analysis of the entire Rad18 gene was not evaluated. They used only genomic DNA extracted from a formalin embedded lung cancer tissue which was PCR amplified and checked only the status of codon 302 SNP and concluded that this SNP is the risk of lung cancer development. The total number of the lung cancer sample was quite large and the frequency of SNP and lung cancer development was statistically different. If this single nucleotide change (which changes the amino acid sequence) is the cause of lung cancer, this is no more a "polymorphism" but a "mutation". And if this nucleotide change is a "mutation", there should be a difference in the function between these two different proteins. Based on the function of Rad18, as a key protein of PRR system, the sensitivity to the DNA damaging reagents (cisplatin and CPT-11) were examined according to the reports [19,20]. Furthermore, when Rad18 is null, it is reported that the growth of the cells won't change but the abnormal morphologies with nuclear segregation will occur [21,22]. Thus we investigated the differences of cell morphology, cell growth and sensitivity to anti-drug agents. Unfortunately, we could not find a difference from both clinical samples and in vitro study. Furthermore, no difference was observed in DNA repair function. Different to the report, we used mRNA and analyzed the whole open reading frame of *Rad18 *gene and also examined the expression level, in vitro analysis.

## Conclusion

From all these results, we came to a conclusion that, there is no relation between Rad18 and lung cancer development. Still there is a possibility that PRR system might be involved in cancer development. As Rad18 interacts with Rad6 and function as a ubiquitin enzyme to activate PCNA, if these key proteins were involved in cancer, the PRR system will not function and might lead to cancer development. Further analysis of this system is required to clear whether there is a relation between PRR and cancer development.

## Competing interests

The authors declare that they have no competing interests.

## Authors' contributions

TN was involved in the molecular genetic study, immunoassays, sequence alignment and statistical analysis. SI was involved in the molecular genetic study, immunoassays, sequence alignment, design of the study, conception of the study and drafting of the manuscript. YK and YN contributed to the molecular genetic study. KI, TM and HN operated and collected the clinical samples. HB: conceived the study and helped to draft the manuscript. All authors read and approved the final manuscript.
